# Supplementary material for: Facilitation of Bone Healing Processes Based on the Developmental Function of Meox2 in Tooth Loss Lesion
Source: Int J Mol Sci. 2020 Nov 18;21(22):8701. doi: 10.3390/ijms21228701 (PMC7698889; doi:10.3390/ijms21228701)
Supplement: Supplementary file 1 [file ijms-21-08701-s001.pdf]

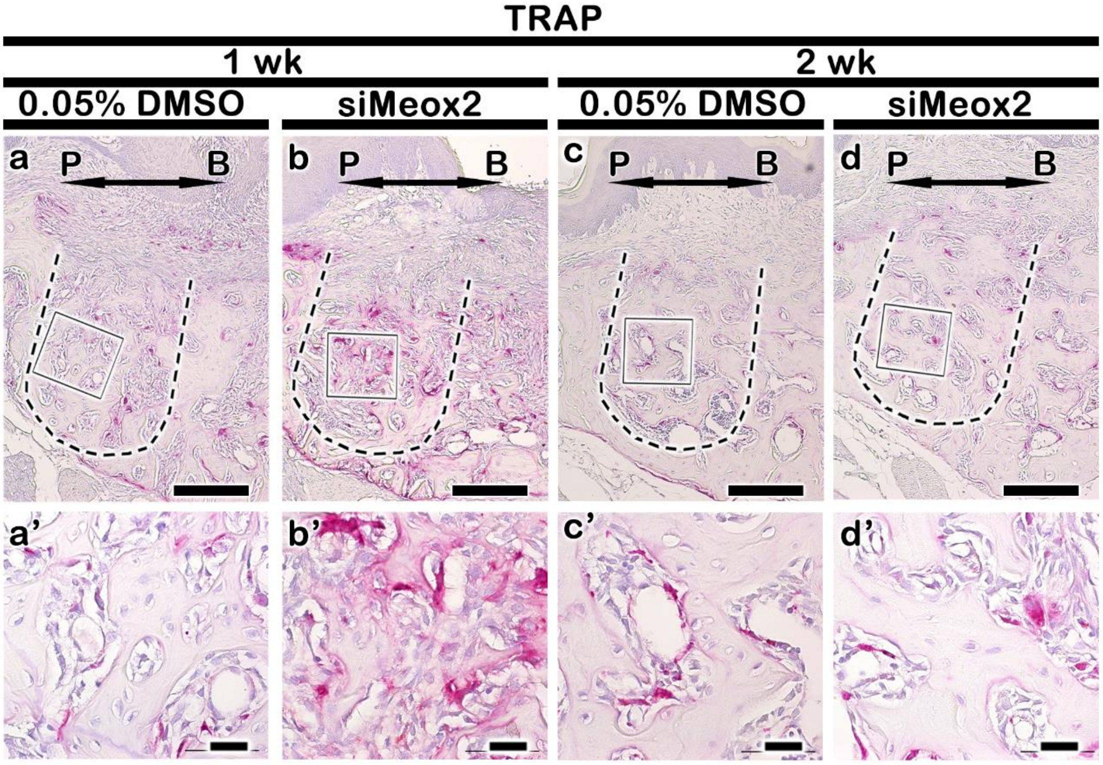

Figure S1. Osteoclast numbers during bone formation. The carrier-control-treated apecimens show higher number of TRAP-positive cells compared to siMeox2 treated specimens (a-d). After 2 weeks of treatment, TRAP-positive cells is higher in the carrier control-treated specimens than siMeox2 treated specimens (c, d). The solid boxes depict higher magnification views (a’-d’). Scale bars 200 µm (a-d), 100 µm (a’-d’).

Table S1. Number of positive pre-osteoblast cells. Dotted box dermacates the 100 μm^2 area used for statistical analysis (n = 12). The table of analysis determined using *, p < 0.05.

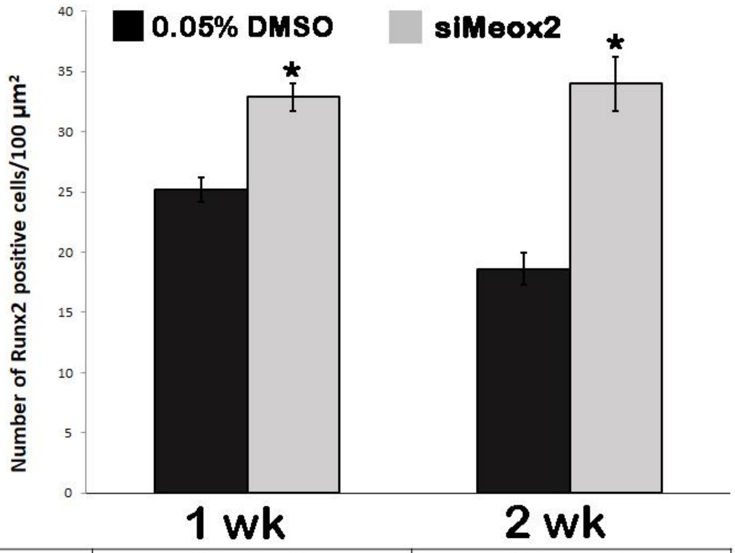

n=12	1 week		2 week	
	DMSO	siMeox2	DMSO	siMeox2
Mean	**25.2**	**32.9**	**18.6**	**34**
STDEV	**2.1**	**2.3**	**2.7**	**4.6**

Table S2. Number of mice used for periodontitis induction.

Number of periodontitis induced mice; (n) = number of mice for Micro-CT		
**Period**	**0.05% DMSO**	**100 nM siMeox2**
1 week	4	4
2 week	4(3)	4(3)
3 week	4	4
Total	12(3)	12(3)
